# Clinical Evaluation of Intraoral, In-Lab Scanners and PVS Impression Materials Using STL Superimposition

**DOI:** 10.3390/dj13120575

**Published:** 2025-12-03

**Authors:** Nader Abdulhameed, Inessa Slipak, Alexandra Manibo, Hind Hussein, Raj Gohel, Emmanouil-George Tzanakakis, Panagiotis Zoidis

**Affiliations:** 1 Restorative Dental Sciences, College of Dentistry, University of Florida, Gainesville, FL 32610, USA; hinds@ufl.edu (H.H.); rgohel@ufl.edu (R.G.); pzoidis@ufl.edu (P.Z.); 2 School of Dentistry, Lake Erie College of Osteopathic Medicine, Erie, PA 16509, USA; 3 Independent Researcher, 15561 Athens, Greece; tzanakak@dent.uoa.gr

**Keywords:** intraoral scanner, in-lab scanner, Polyvinylsiloxane, STL files, dental impression materials

## Abstract

**Background/Objective.** The objective of this study is to test the hypothesis that there is no significant difference between intraoral scanners and in-lab scanners. An additional objective is to test the hypothesis that there is no significant difference between the accuracy of two types of polyvinyl siloxane PVS impression materials and between PVS impression materials and intraoral scanners. **Material and Methods.** Fourteen subjects received a set of maxillary and mandibular removable complete dentures [RCD]. Impressions of each RCD were obtained using two PVS impression materials (heavy [H] or medium [M] body with light [L] body). Each RCD was then scanned utilizing two intraoral scanners, Trios [TR] and Omnicam [OM]. The PVS impressions were sent to the lab to be further scanned by an in-lab scanner. STL files of the intraoral and in-lab scans were obtained and trimmed using the GeoMagic X Software. The files were merged [TR vs. OM] and [TR vs. in Lab], [HL vs. ML], [HL vs. TR] and [ML vs. TR] and the gap was measured in sixty points for each merged file. **Results.** There was no significant difference between [TR vs. OM] with a mean of 44 ± 10 μm. There was a statistically significant difference between [TR vs. In lab] with a mean of 62 ± 21 μm and [ML vs. TR] and [HL vs. ML] with means of 66 ± 24 μm and 50 ± 21 μm, respectively. There was no significant difference between [HL vs. TR] with a mean of 37 ± 10 μm. **Conclusions.** Intraoral scanners provide a similar quality of scans. The lab scanner depends on the impression materials used. The first null hypothesis was rejected. Using a heavy and light body PVS impression material provides more accurate and dimensionally stable impressions, particularly in full-arch applications, and is comparable to intraoral scanners. The second null hypothesis was rejected, there were significant differences between the PVS groups. These findings guide clinicians in selecting impression methods for complete dentures.

## 1. Introduction

Dental impression materials are used to replicate exact hard and soft tissue structures intraorally. The accuracy of the replication, dimensional stability, technique used to capture the intraoral structures, reproducibility, and physical and chemical properties all play a key role in determining what kind of impression material to use. There have been many advancements over the years that have improved material properties such as taste and setting time; however, the process of impression making is time-consuming for the dentist and uncomfortable for patients, especially for those with a gag reflex [1,2].

Conventional impression materials can be divided into two types based on their biomechanical properties: non-elastic and elastic. Non-elastic impression materials exhibit an insignificant amount of elastic deformation [3]. Elastic impression materials, on the other hand, are rubbery polymers that are cross-linked, with the advantage of accurately reproducing both soft and hard tissues even in the presence of undercuts. These are the most used impression materials for edentulous patients [4].

In a dental setting, obtaining accurate diagnostic records with conventional impression materials is considered the gold standard of care mainly due to their cost-effectiveness [5]. Traditional impression techniques, however, place a substantial burden on dental professionals to accurately create restorations that match the tooth surface(s). Conventional impressions are more prone to accumulating errors because of the properties of dental materials [6]. The multiple steps involved in handling impression materials by the dentist and dental laboratory introduce errors that affect the final quality and delivery of the restorations.

Today, polyvinyl siloxane (PVS) is the most widely used elastomer in dental practice. The advantages of using PVS as an impression material are attributable to its low dimensional change, low creep, shorter setting time, and tear resistance [7]. The absence of by-products during the polymerization reaction gives the impression of dimensional stability, which allows it to be poured at the convenience of the operator [8].

Digital dentistry has introduced new technologies that enable the creation of a reproducible 3D image of the scanned area, which is used to design a prosthesis. Digital impressions have been introduced in multiple aspects of dentistry, with their primary benefit being the avoidance of errors that can occur when using conventional dental materials [9]. One of the benefits of digital impressions is the reduction in time expenditure. Other benefits of using an intraoral optical scanner (IOS) for digital impressions include the ease of use, which eliminates the need for a tray, dispensing, and polymerization of material, as well as the costs and time associated with laboratory shipping, all while minimizing patient discomfort [2,10]. It is essential to recognize that intraoral scanning accuracy is influenced by several factors, including scanning strategy, operator experience, ambient lighting, and patient-related variables such as saliva and patient movement. These factors can introduce variability in the final STL output. Fratila et al. (2025) provide a comprehensive overview of these influences and propose procedural guidelines to enhance IOS accuracy [11].

Intraoral scanners enable us to accurately capture impressions of the upper and lower arches using only a light beam. The impression data is imported into a software program, which converts it into a standard triangle language (STL) file. This STL file is then interpreted by the scanners in the dental laboratory, and the model is physically created and then delivered to the patient [12]. Using Computer-Aided Design and Computer-Aided Manufacturing (CAD/CAM) for short-span digital impressions minimizes the risk of error compared to when it’s used for greater areas in the oral cavity. The precision is decreased as you utilize CAD/CAM for areas larger than a quadrant [13]. From our investigation of other studies, results of only a few studies that investigated the trueness of fully edentulous and partially edentulous models concluded that the longer the scan range, the larger the error [14]. This limitation is particularly relevant to our study, which involves full-arch impressions of complete dentures, where scanning larger areas may introduce cumulative errors.

The primary research question of this study is: Is there a significant difference in accuracy between intraoral scanners, in-lab scanners, and various PVS impression material combinations when used for full-arch impressions? This comparison is essential because it helps clinicians identify the most accurate and efficient method for capturing full-arch impressions, which directly influences prosthesis fit and patient satisfaction.

Intraoral scanners capture impressions directly from the patient’s mouth, in-lab scanners digitize physical models, and extraoral scanners refer to lab-based devices used for indirect scanning.

Digital impressions with intraoral scanners must be comparable to using conventional impressions to achieve clinical success [15]. Extraoral scanners comprise an impression and a scan of the impression. Extraoral scanners are designed to either scan the impression directly and create a virtual die or scan a stone die [16]. The use of impressions for extraoral scanners yields its fair share of errors, including accuracy of the impression or model, material used to pour the model, water retention, and reproducibility, among others.

‘Accuracy’ as defined by the International Organization for Standardization standard 5725-1 [17], encompasses both trueness and precision. ‘Trueness’ is the measured deviation from the actual value or dimension of the object. ‘Precision’ is defined as a measure of repeatability or how close a set of results are to each other [18]. Trueness refers to the closeness of a measurement to the actual value and precision expresses the degree of reproducibility between repeated measurements [6]. Considering rapid advancements in digital dentistry, the recent literature has been incorporated to reflect current scanner technologies and material performance. Studies from 2022 to 2025 were prioritized to ensure relevance to contemporary clinical practice.

Given the increasing adoption of digital workflows and the continued use of conventional materials, it is essential to evaluate their comparative performance under controlled conditions. This study uniquely evaluates scanner accuracy using extraoral scans of complete dentures, avoiding intraoral variables.

The objectives of this study are to test the first null hypothesis that there is no significant difference between intraoral scanners and in-lab scanners using PVS impression materials and to test the second null hypothesis that there is no significant difference between the accuracy of two PVS impression materials after scanning with in-lab scanners.

## 2. Materials and Methods

Fourteen subjects received a set of upper and lower complete removable dentures through the Clinical Complete Denture Course. The participants were edentulous patients aged 58–76 years (mean age, 66.4), with no significant oral pathology, who were enrolled in the Clinical Complete Denture Course.

After obtaining approval from the Institutional Review Board (IRB) protocol (26-157), informed consent was obtained prior to treatment, and an agreement to participate in the study was signed. All treatment steps in the fabrication and delivery of the complete dentures. At the 1-week recall appointment post-denture delivery. The upper and lower complete dentures were scanned using two intraoral scanners: Trios 4 (3Shape, Copenhagen, Denmark; TRIOS Software 19.2) [TR] and CEREC/Sirona Omnicam (Dentsply Sirona, Charlotte, NC, USA; CEREC SW 5.1) [OM] acquisition unit. All scans were performed by a single calibrated operator in a controlled environment to minimize variability. The scanner was moved in a continuous, overlapping motion to ensure complete data capture, following the manufacturer’s recommended protocols. Occlusal, lingual, then buccal.

Although impressions were made on dentures outside the mouth to improve control, this limitation restricts the clinical validity of the study. No power calculation was performed due to the exploratory nature of the study. Intraoral scanners capture impressions directly from the patient’s mouth, in-lab scanners digitize physical models, and extraoral scanners refer to lab-based devices used for indirect scanning.

**Figure 1:**
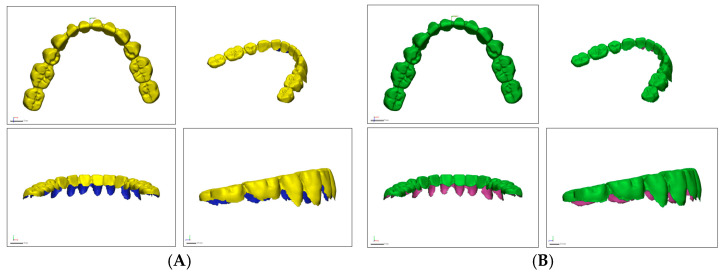
Trimmed STL files. (**A**), Trios Intraoral Scanner. (**B**), In-Lab Scanner.

In this study, our primary concern was to capture the occlusal surfaces of the dentures, rather than the acrylic bases. PVS impressions of the upper and lower complete dentures were obtained. Heavy body with light body [HL] PVS impression materials, Virtual XD (Ivoclar Vivadent, Schaan, Liechtenstein) and medium body with light body [ML], Aquasil (Dentsply Sirona, York, PA, USA) were utilized to obtain impressions of the upper and lower dentures extraorally (Figure 1).

First, a heavy body was injected onto a piece of white paper; one for the maxillary complete denture and one for the mandibular complete denture. Simultaneously, the light body (wash impression material) was injected onto the maxillary complete denture and mandibular complete denture occlusal surfaces. A heavy body material was placed on paper to simulate tray support while minimizing distortion, as determined through pilot testing. The dentures were placed on their respective heavy body impression material, and the impressions were allowed to set according to the manufacturer’s instructions. Similarly, two mounds of medium body were injected onto the white paper, with light body injected on the occlusal surfaces of the maxillary and mandibular complete dentures. They were then allowed to set according to the manufacturer’s instructions.

To standardize the amount of PVS, impressions only required occlusal and incisal surfaces of denture teeth with similar-sized molds for all dentures. Following the manufacturer’s instructions, the PVS impressions were carefully stored in a box and transported to the lab, where extraoral digital impressions were obtained using an in-lab scanner E4 scanner (3Shape, Copenhagen, Denmark; Dental System 2019). The digital impressions of the intraoral and in-lab scans were converted to standard triangle language (STL) format with no patient identifiers attached to the files.

The files obtained with the intraoral and extraoral scanners were converted to standard triangle language (STL) format. Discrepancies (standard deviation) were analyzed using the Geomagic Control X (3D Systems, Rock Hill, SC, USA; version 2020.0.0) by selecting various parameters of the files (intraoral scanners: [TR vs. OM], [TR vs. In Lab], PVS impression material with intraoral scanner: [HL vs. ML], [HL vs. TR] and [ML vs. TR]).

**Figure 2:**
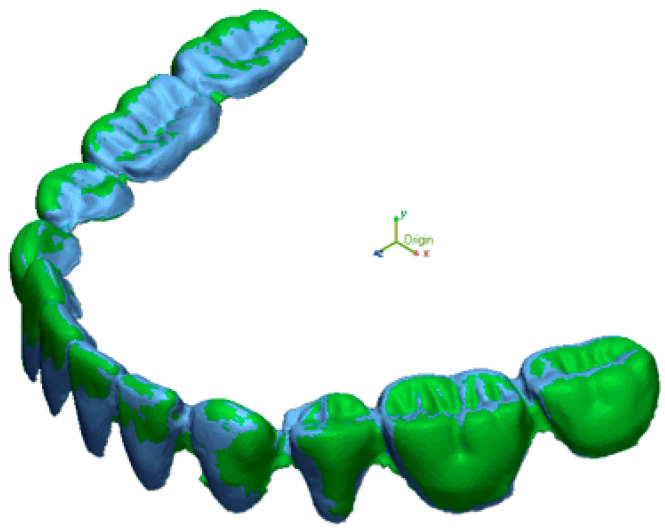
Superimposition of trimmed STL files of TR and OM using “best fit alignment” feature on Geomagic.

With the “cut the planes” tool, all the soft tissue (acrylic base) surrounding the teeth was trimmed up to the gingival margin, leaving the interdental papilla at the level of the junction of the middle and cervical one-third to focus the data points on selected areas on the teeth (Figure 2)

**Figure 3:**
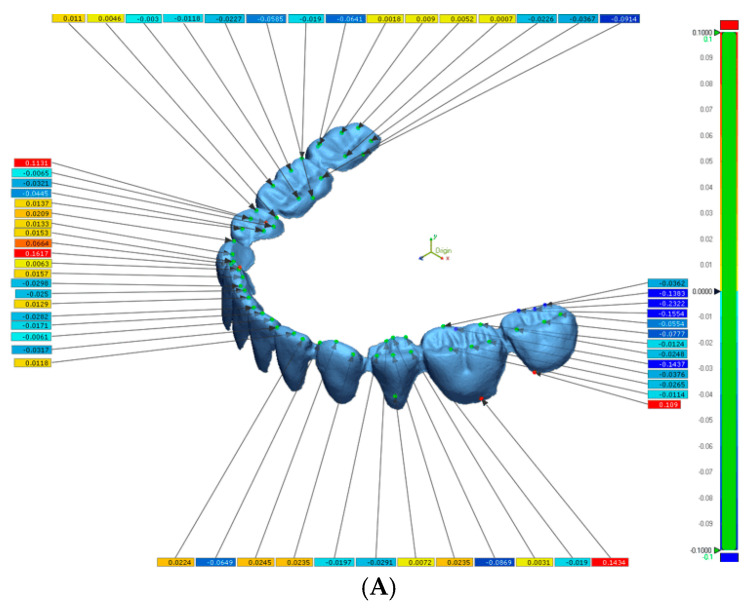

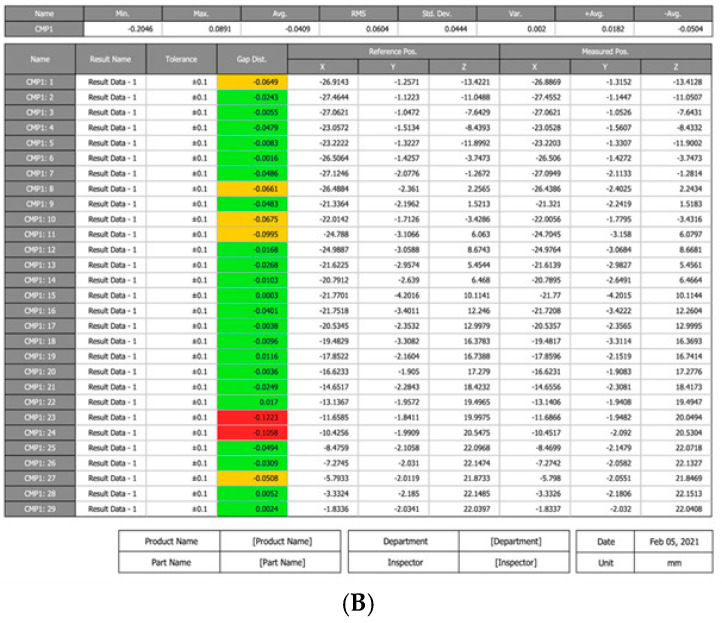
Images of Geomagic Report. (**A**), Selection of 60 data points of merged STL files. (**B**), Values for statistical analysis.

The STL files of Trios were used as the reference file and those of the Omnicam and In-Lab STL files were selected as the measured file. The data points selected for comparison were based on the cusps of the denture teeth. The measured files were superimposed to the reference file to calculate the total deviations between the selected data from the reference scanner and the measured scanners (Figure 2). The resolution was set to high (0.01 mm), and the “best fit alignment” algorithm was used to superimpose STL files. Sixty data points were manually selected on cusp tips and incisal edges to ensure consistency across comparisons. (Figure 3).

**Figure 4:**
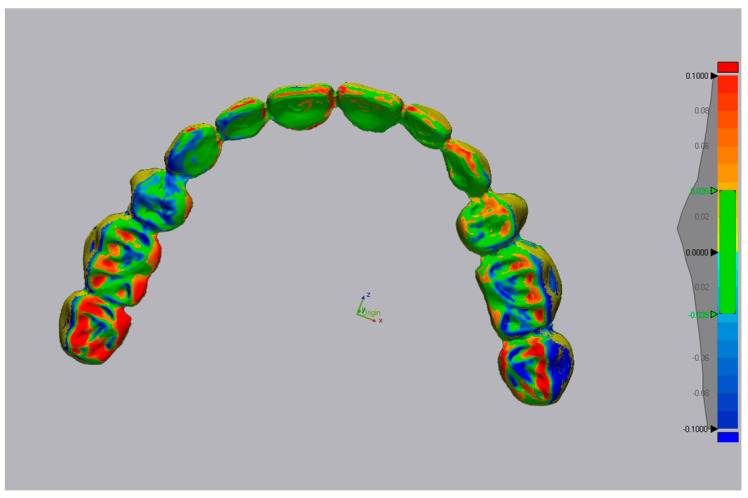
Color-coded superimposed image. Red color (expansion), Blue color (contraction).

The deviation is measured in micrometers; both positive (expansion) and negative (contraction) are viewed on a color-coded superimposed image (Figure 4) [12].

## 3. Results

**Figure 5:**
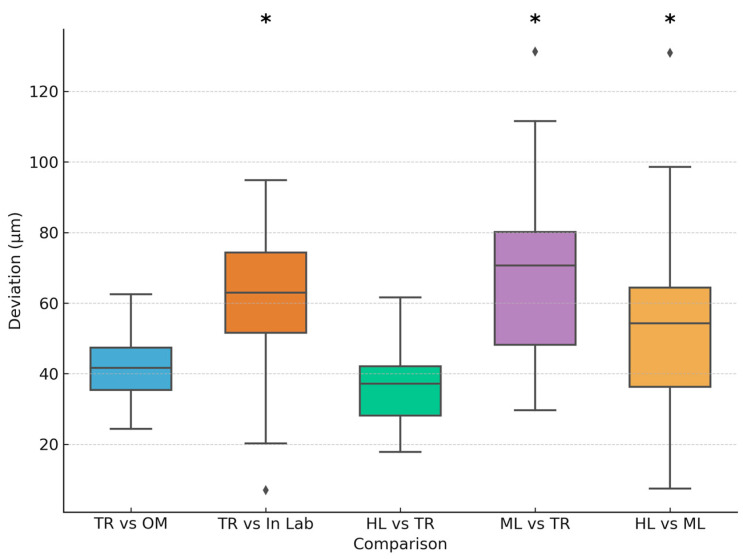
Mean and standard deviation for group comparison, significance markers (*). The (⧫) markers represent outliers in the dataset.

After performing One-way ANOVA and Tukey HSD tests, there was no significant difference between [TR vs. OM] with a mean deviation of 44 ± 10 μm. There was a statistically significant difference (*p* < 0.05) between [TR vs. In lab] with a mean deviation of 62 ± 21 μm. There was no significant difference between [HL vs. TR] with a mean deviation of 37 ± 10 μm. There was a statistically significant difference (*p* < 0.05) between [ML vs. TR] and [HL vs. ML] with mean deviations of 66 ± 24 μm and 50 ± 21 μm, respectively. ML was the only group that showed significant differences. The ML group showed statistically significant deviations in all comparisons it was involved in, indicating a consistent pattern of lower accuracy. Table 1. and Figure 5. Summarizes the results of one-way ANOVA and Tukey HSD tests comparing different scanner and material combinations.

## 4. Discussion

The present study analyzed the accuracy of intraoral and laboratory scanners, as well as various PVS impression materials. Based on our research, there are very few articles that compare the precision and trueness of extraoral scanners with those of intraoral scanners in regard to full-arch digital impressions. The results obtained were then compared to see if there were any significant differences between the impression and scanning techniques. There were no significant differences when comparing the intraoral scanners themselves or when using them in conjunction with the in-lab scanners. There were differences in accuracy when comparing the medium body-light body to the Trios scanner as well as comparing HL to ML. 

The accuracy of the dental impression is crucial for the overall performance of the restoration or prosthesis when it is below 100 µm. The ability to capture the hard and soft tissues in the most accurate way reduces potential errors during the fabrication process. Several materials and techniques are available to enhance the accuracy of the impression. Multiple PVS impression materials are available as well as digital scanners for taking impressions. The PVS material exhibits excellent physical properties, including elastic recovery, the ability to record fine details, and dimensional stability [18,19,20,21]. The PVS materials also come in different viscosities, which are incorporated into various techniques. Each technique should yield accurate results with minimal deviation. To test this hypothesis, the study was conducted to compare the PVS impression techniques with each other, as well as with digital impressions taken using TR and OM.

A recent study was conducted to compare the viscosities of PVS impression materials by examining their dimensional accuracy [22]. In their study, the viscosities and techniques involved the following: putty, medium body, medium body with light body, and heavy body with light body. Impressions were made on a dentulous acrylic resin denture base with 6 natural teeth. The 6 Teeth were prepped for a crown. Specific reference points were chosen and measured to analyze the accuracy of the materials. According to the study, the results revealed that the heavy body and the light body yielded more accurate results compared to other techniques. However, all the materials showed acceptable accuracy within clinical parameters. Comparing these results to our study revealed similarities when a medium body was incorporated into the impression technique. In this study, the medium body with light body showed significant deviation compared to the heavy body and light body impressions.

In a previous study comparing conventional and digital impression techniques, the conventional PVS impression was significantly different than the digital impressions taken [21]. Additionally, the study found that the two intraoral scanners being compared (Trios vs. Omnicron) did not exhibit significant differences, which aligns with our findings. Malik et al. [23] used a reference model to obtain and compare the various impression techniques. They used additional silicone for the conventional impression material. Similarly to our study, Geomagic was used to superimpose the scans in order to evaluate trueness and precision. A key difference between this study and ours is that the researchers selected a limited number of teeth for comparison: the palatal and incisal areas of the anterior teeth and the occlusal third of the second molars. The current study evaluated every denture tooth to assess any deviation between impression techniques. Additionally, this study selected points of occlusal contact for comparison of deviations rather than entire tooth surfaces. However, despite these differences in methodology, the previous study yielded similar results to the current study.

Both scanners, Trios and Omicron, capture thousands of pictures that are processed into a 3D image by scanning software. The scanners relied on a light to reveal the areas its scanning. This reflective property of the tissue or material being scanned may alter the final 3D image created. A new scannable impression material is now available that can reduce errors caused by light reflection. A recent study compared the accuracy of digitization of scannable and non-scannable elastomeric impression materials. A master die in the shape of a ceramic crown prep was used and digitized for a reference. Impressions of the master die were made using scannable and non-scannable materials. The study’s results revealed that areas exposed to the scanner’s light were digitized more accurately with the scannable material. For both materials, the lowest amount of discrepancy was the margins and axial walls [24]. The use of scannable impression material could be incorporated into this current study to see if other observations could be made. Other characteristics, such as color, surface texture, and morphology must also be taken into consideration when using digital scanners and the type of impression material.

While earlier studies such as Flugge et al. (2013) [11] provided foundational insights, they do not reflect the capabilities of current-generation scanners. Recent evaluations [25,26] demonstrate that modern intraoral scanners like Trios and Omnicam have significantly improved in terms of trueness and precision. Therefore, conclusions in this study are now contextualized using up-to-date evidence.

Ender et al. compared the accuracy and conventional and digital methods of obtaining full arch dental impressions. A steel reference model was made from a patient’s maxillary impression. The reference model had known morphology and was used to compare the various impression techniques. The authors examined eight different conventional materials and four digital impression techniques. The results revealed that the conventional materials had higher trueness and precision compared to the digital impressions. When analyzing the digital impressions, they had higher local deviations of the full arch model, revealing less accuracy compared to conventional impressions [27,28]. However, when comparing this study to our current study, the use of a steel reference model could provide a source of error when taking the impressions. The impression materials may behave differently when capturing the surface of the steel model. Also, for the digital impression, the scanner may behave differently when trying to capture the metal surface. In our study, the impressions were used on maxillary and mandibular dentures, which could play a factor in the final results for each impression technique.

As stated previously, the length of the area plays a role in the results of each impression [13]. Kuhr et al. examined a new method that assesses the accuracy of full-arch impressions. The authors used reference spheres placed at specific dimensions on the arch. The measurements were made using a high-precision coordinate measuring machine. Conventional impressions were poured up into casts and the digital models were analyzed using Inspection Software [29]. Based on the study’s results, the conventional impression material showed lower deviations for all recorded distances. The largest deviations for all impression techniques were the intermolar distances. The longer the span for the impression, the greater the deviation observed.

Only two intraoral scanners (Trios and Omnicam) and two PVS material combinations (HL and ML) were evaluated in this study. Other scanners or impression materials might yield different results and could influence the accuracy outcomes. Although clinical impressions were used, the scanning and merging process was performed under controlled conditions, which may not fully replicate intraoral challenges such as saliva, patient movement, or limited access. These factors could affect the performance of intraoral scanners in real clinical settings. Future studies should aim to correlate digital accuracy with clinical outcomes such as denture fit, retention, and patient satisfaction. Additionally, incorporating real-time intraoral scanning conditions would better simulate clinical challenges and provide more clinically relevant insights. These findings suggest that while intraoral scanners perform consistently regardless of brand, the choice of impression material significantly affects the accuracy of lab-scanned models. The ML combination introduced greater variability, possibly due to its lower viscosity and flow characteristics. The superior performance of the HL combination may be attributed to the rigidity of the heavy body material, which stabilizes the light body during setting, reducing distortion.

Our results align with recent evidence emphasizing the importance of scanner calibration and procedural factors in achieving optimal accuracy. Fratila et al. [30] highlighted that scanning strategy and operator technique significantly influence IOS performance, which supports our controlled scanning protocol. Similarly, Kanmaz et al. [26] demonstrated that TRIOS 3 accuracy improves after calibration, reinforcing the reliability of our IOS findings. Advances in IOS technology, as reported by Eggmann and Blatz [31] and comparative analyses by Ciocan et al. [25], confirm that modern scanners deliver clinically acceptable accuracy for full-arch impressions, consistent with our observation that IOS accuracy is comparable to HL PVS. Furthermore, Singer et al. [32] emphasized the dimensional stability of elastomeric materials, which explains the superior performance of HL PVS in our study.

The results of this study can be considered by clinicians when selecting the appropriate impression technique for a specific dental procedure. It also draws attention to the type of materials being used and how lab scanners may yield varying results depending on the material sent to the lab for scanning. The intraoral scanners did not have significant differences, while the lab scanners depended on the material type being scanned.

High standard deviations indicate variability in scanner performance and impression consistency, which should be considered when interpreting mean values. Within the limitations of this exploratory study, HL PVS impressions demonstrated accuracy comparable to intraoral scanners under controlled conditions; however, clinical validation is needed. Intraoral scanners offer a reliable alternative, particularly when patient comfort or time efficiency is a priority. However, material selection remains critical when impressions are sent for lab scanning. Limitations include the small sample size, the use of power analysis from our wear study for the full arch, the use of denture models instead of intraoral scans, and the exclusion of real-time intraoral variables, such as saliva and patient movement, that need to be considered. Future studies should evaluate the clinical fit of prostheses derived from each technique and explore additional scanner and material combinations under in vivo conditions.

## 5. Conclusions

Within the limitations of the study, the intraoral scanners provided a similar quality of digital impression. The in-lab scanner had a statistically significant deviation compared to the intraoral scanner when included in the ML group. The ML group exhibited the highest deviation, highlighting the importance of material selection in achieving accurate impressions.

The first null hypothesis was rejected. Using a combination of heavy and light body PVS impression materials provides more accurate and dimensionally stable impressions, particularly in full-arch applications. The second null hypothesis was rejected, and there were significant differences between the PVS groups.

## Figures and Tables

**Table 1:** Mean deviations and statistical significance (*p*-values) for scanner and impression material comparisons.

Comparison	Mean ± SD (µm)	Significance	Exact *p*-Value
TR vs. OM	44 ± 10	Not Significant	0.412
TR vs. In Lab	62 ± 21	Significant	0.018
HL vs. TR	37 ± 10	Not Significant	0.276
ML vs. TR	66 ± 24	Significant	0.021
HL vs. ML	50 ± 21	Significant	0.033

## Data Availability

The original data supporting the findings of this study are stored securely by the authors and are available upon reasonable request. Further inquiries can be directed to the corresponding author.
